# Corrigendum: Recombination spot identification Based on gapped k-mers

**DOI:** 10.1038/srep35331

**Published:** 2016-12-07

**Authors:** Rong Wang, Yong Xu, Bin Liu

Scientific Reports 6: Article number: 2393410.1038/srep23934; published online: 03 31 2016; updated: 12 07 2016.

This Article reports an application of methodology originally reported in Reference 33 to recombination spot identification. Reference 33 of this Article introduced a feature set called gapped k-mer for regulatory sequence prediction; this Article applied these gapped k-mer features to recombination spot identification, and a computational predictor was constructed for recombination spot identification.

In the original version of the Article, the Abstract included ambiguous sentences which failed to give due credit to the authors of Reference 33. The authors apologize for these errors.

“The k-mer feature is one of the most useful features for modeling the properties and function of DNA sequences. However, it suffers from the inherent limitation. If the value of word length *k* is large, the occurrences of k-mers are closed to a binary variable, with a few k-mers present once and most k-mers are absent. This usually causes the sparse problem and reduces the classification accuracy. To solve this problem, we add gaps into k-mer and introduce a new feature called gapped k-mer (GKM) for identification of recombination spots. By using this feature, we present a new predictor called SVM-GKM, which combines the gapped k-mers and Support Vector Machine (SVM) for recombination spot identification. Experimental results on a widely used benchmark dataset show that SVM-GKM outperforms other highly related predictors. Therefore, SVM-GKM would be a powerful predictor for computational genomics”.

now reads:

“k-mer is one of the commonly used features for recombination spot identification. However, when the value of *k* grows larger, the dimension of the corresponding feature vectors increases rapidly, leading to extremely sparse vectors. In order to overcome this disadvantage, recently a new feature called gapped k-mer was proposed (Ghandi *et al.*, *PloS Computational Biology*, 2014). That study showed that the gapped k-mer feature can improve the predictive performance of regulatory sequence prediction. Motived by its success, in this study we applied gapped k-mer to the field of recombination spot identification, and a computational predictor was constructed. Experimental results on a widely used benchmark dataset showed that this predictor outperformed other highly related predictors”.

In addition, there were errors in the definition of 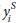 in Equation 2.

“where 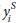 is the length of the *i* − *th* gapped k-mer in the sequence S,”

now reads:

“where 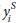 is the count of the *i* − *th* gapped k-mer in the sequence S,”

There were errors in the definition of *r* in Equation 6.

“The remaining mismatches *r* = *n*_2_ − *t* − (*n* − *n*_l_ + *t*) are among the the *n* mismatch positions for k-mer *x*_2_”.

now reads:


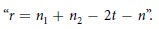


The following sentence has been added to the end of the first paragraph in the ‘Gapped k-mer’ section:

“Eqs 2–6 were originally reported in ref. 33. For further explanation of these equations, please refer to ref. 33”.

There were errors in Equation 7.
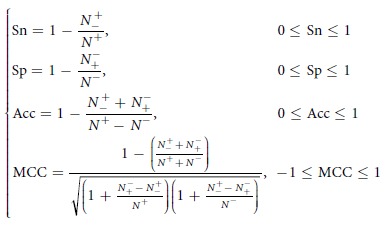


now reads:
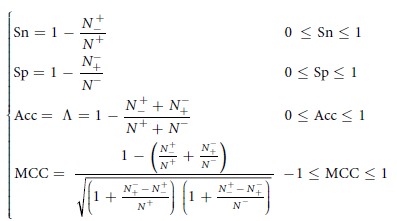


Equation 7 also appears in Reference 1 and in Reference 65.

These errors have now been corrected in the HTML and PDF versions of this Article.

